# Early short-term hypoxia promotes epidermal cell migration by activating the CCL2-ERK1/2 pathway and epithelial–mesenchymal transition during wound healing

**DOI:** 10.1093/burnst/tkae017

**Published:** 2024-06-17

**Authors:** Linbo Jin, Shiqi Zhou, Shihan Zhao, Junhui Long, Zhidan Huang, Junli Zhou, Yiming Zhang

**Affiliations:** Department of Plastic and Cosmetic Surgery, Xinqiao Hospital, Army Medical University, Chongqing, 400037, China; Department of Plastic and Cosmetic Surgery, Xinqiao Hospital, Army Medical University, Chongqing, 400037, China; Department of Plastic and Cosmetic Surgery, Xinqiao Hospital, Army Medical University, Chongqing, 400037, China; Department of Dermatology, Southwest Hospital Jiangbei Area (The 958th hospital of Chinese People’s Liberation Army), Chongqing, China; Department of Plastic and Cosmetic Surgery, Xinqiao Hospital, Army Medical University, Chongqing, 400037, China; Department of Burn and Plastic Surgery, The Tenth Affiliated Hospital of Southern Medical University (Dongguan People's Hospital), Dongguan, China; Department of Plastic and Cosmetic Surgery, Xinqiao Hospital, Army Medical University, Chongqing, 400037, China

**Keywords:** Hypoxia, Epidermal cells, Wound healing, Epithelial–mesenchymal transition, CCL2, Extracellular signal-regulated kinase

## Abstract

**Background:**

Due to vasculature injury and increased oxygen consumption, the early wound microenvironment is typically in a hypoxic state. We observed enhanced cell migration ability under early short-term hypoxia. CCL2 belongs to the CC chemokine family and was found to be increased in early hypoxic wounds and enriched in the extracellular signal-regulated kinase (ERK)1/2 pathway in our previous study. However, the underlying mechanism through which the CCL2-ERK1/2 pathway regulates wound healing under early short-term hypoxia remains unclear. Activation of epithelial–mesenchymal transition (EMT) is a key process in cancer cell metastasis, during which epithelial cells acquire the characteristics of mesenchymal cells and enhance cell motility and migration ability. However, the relationship between epithelial cell migration and EMT under early short-term hypoxia has yet to be explored.

**Methods:**

HaCaT cells were cultured to verify the effect of early short-term hypoxia on migration through cell scratch assays. Lentiviruses with silenced or overexpressed CCL2 were used to explore the relationship between CCL2 and migration under short-term hypoxia. An acute full-thickness cutaneous wound rat model was established with the application of an ERK inhibitor to reveal the hidden role of the ERK1/2 pathway in the early stage of wound healing. The EMT process was verified in all the above experiments through western blotting.

**Results:**

In our study, we found that short-term hypoxia promoted cell migration. Mechanistically, hypoxia promoted cell migration through mediating CCL2. Overexpression of CCL2 via lentivirus promoted cell migration, while silencing CCL2 via lentivirus inhibited cell migration and the production of related downstream proteins. In addition, we found that CCL2 was enriched in the ERK1/2 pathway, and the application of an ERK inhibitor *in vivo* and *in vitro* verified the upstream and downstream relationships between the CCL2 pathway and ERK1/2. Western blot results both *in vivo* and *in vitro* demonstrated that early short-term hypoxia promotes epidermal cell migration by activating the CCL2-ERK1/2 pathway and EMT during wound healing.

**Conclusions:**

Our work demonstrated that hypoxia in the early stage serves as a stimulus for triggering wound healing through activating the CCL2-ERK1/2 pathway and EMT, which promote epidermal cell migration and accelerate wound closure. These findings provide additional detailed insights into the mechanism of wound healing and new targets for clinical treatment.

Highlights

- In the early stage of wound formation, hypoxia induces high expression of CCL2, which is enriched in the ERK1/2 pathway.
- Silencing/overexpressing CCL2 via lentivirus causes impaired/enhanced cell migration under early short-term hypoxia.
- HaCaT cells acquire migratory ability through the epithelial–mesenchymal transition process under early short-term hypoxia.
- An ERK inhibitor causes decreased cell migration and delayed wound healing in an acute full-thickness cutaneous wound rat model.

## Background

The skin is the largest barrier for humans to harmful external factors, such as ultraviolet radiation, bacteria and mechanical damage, which also increase vulnerability to injuries [1]. After suffering an injury, wound healing follows a dynamic and well-organized process with great complexity that consists of three overlapping stages: inflammation, proliferation and remodeling [2, 3]. The main goal of current treatments is to promote wound closure, in which the migration of epidermal cells is the initial event and the speed-limiting step, and it is also an important stage in which re-epithelialization is completed [4, 5]. The loss of migration ability and consequent incomplete re-epithelialization cause delayed or even failed wound healing [6, 7], which ultimately not only affects the physical and mental health and quality of life of patients but also places serious economic burdens on patients’ families and society [8, 9]. Therefore, how to improve the quality of wound healing has become a problem of practical significance that urgently needs to be solved.

After acute injury to skin tissue, the microenvironment of the wound is in a state of hypoxia or anoxia due to the rupture of blood vessels and the high oxygen consumption of related cells [10]. In the early stage, the hypoxic environment can trigger the secretion of various factors, such as transforming growth factor, vascular endothelial growth factor and platelet-derived growth factor, by cells around the wound [11]. These chemokines regulate the spatiotemporal recruitment of different types of white blood cells and accelerate wound repair to the next stage. Based on these findings, focusing on how the early hypoxia microenvironment initiates the process of wound healing, our previous work with a gene chip system demonstrated markedly greater CCL2 expression in HaCaT cells after 24 h of hypoxia treatment.

CCL2 belongs to the CC chemokine family and can be secreted by various types of cells, such as fibroblasts, vascular smooth muscle cells, endothelial cells, monocytes and T cells [12]. Increasing evidence suggests that CCL2 can recruit monocytes, memory T cells and dendritic cells to inflammatory sites during tissue damage or infection [13]. CCL2 has also been found to be involved in the migration of microglia and mediates the infiltration of T lymphocytes in ischemic stroke brain models [14]. Hypoxia can induce adipose stem cells to promote angiogenesis in human dermal microvascular endothelial cells in flap tissue through the CCL2/CCR2 pathway [15]. CCL2 can also recruit macrophages to accelerate adipose tissue fibrosis [16]. Although the chemotactic effect of CCL2 on other kinds of cells has been confirmed in many disease models, the mechanism by which it affects epidermal cell migration in acute wounds remains unclear.

Through further Gene Ontology (GO) and Kyoto Encyclopedia of Genes and Genomes (KEGG) pathway analyses, we found that CCL2 was enriched in the extracellular signal-regulated kinase (ERK)1/2 pathway. As part of the mitogen-activated protein kinase (MAPK) signaling cascade, ERK typically responds to cellular pressure [17]. In tumor models, hypoxia stimulates ERK in different cell types, which is beneficial for resisting cell apoptosis [18–21]. Hypoxia promotes the migration of bone marrow mesenchymal stem cells and endothelial cells by inducing ERK phosphorylation. In a rat model of pulmonary hypertension, vascular peroxidase 1 promoted the phenotypic transformation of pulmonary artery smooth muscle cells under hypoxic conditions through the ERK pathway [22]. However, the relationships between hypoxia-induced ERK activation and CCL2 expression and epidermal cell migration have not been studied.

Epithelial–mesenchymal transition (EMT) usually occurs during cancer cell metastasis and features the expression of mesenchymal cell markers and loss of epithelial cell markers, indicating the acquisition of migration ability to surrounding tissues [23–25]. EMT is also commonly observed during embryonic development and allows initial embryonic epithelial tissue to transform into mesenchymal cells to migrate and differentiate [26]. EMT has also been reported in the human uterine endometrium, which might constitute a unique wound healing model. Because of menstruation and parturition, the uterine endometrium undergoes repeated remodeling and regeneration, and EMT was found to be able to provide the necessary phenotype and functional flexibility for successful decidualization of the endometrium [27]. However, the role of EMT in hypoxia-triggered cell migration has yet to be explored.

Although the roles of hypoxia, CCL2, ERK and EMT in promoting cell migration have been established in other physiological or pathological models, studies focusing on the relationship between these factors in wound healing are relatively rare and not in depth. In addition, there are also some examples in which the same biomolecule has completely opposite effects on different physiological or pathological models. For instance, trefoil factor family 1 (TFF1) is a peptide that was found to inhibit gastric cancer but promote the progression of breast cancer. Mice with TFF1 deficiency were found to develop antropyloric dysplasia, which is likely to lead to an increase in adenoma and carcinoma [28, 29]. However, high expression of TFF1 was shown to be associated with the estrogen status of patients and was found mainly in bone metastases from breast cancer [30, 31]. In addition, the *myosin heavy chain 9* gene was found to promote the migration of synovial fibroblasts in patients with rheumatoid arthritis and protect the nucleus during cell migration in prostate cancer [32, 33], while the knockdown of *myosin heavy chain 9* was linked to the invasion and distant metastasis of cancer cells [34, 35].

In this study, we focused on the underlying mechanism by which the CCL2-ERK1/2 pathway affects epidermal cell migration and accelerates wound healing under short-term hypoxia and how epidermal cells acquire migratory ability. Both *in vivo* and *in vitro* experiments have indicated that short-term hypoxia conditions can serve as a switch for the CCL2-ERK1/2 pathway, thereby promoting wound healing. The application of lentivirus transfection and ERK inhibitors elucidated the roles of the CCL2 and ERK pathways in early short-term hypoxia-induced epidermal cell migration and clarified the upstream and downstream relationships, respectively. Western blot (WB) results confirmed the occurrence of EMT. In addition, injection of an ERK pathway inhibitor into the wounds of rats also delayed wound healing, decreased the expression of CCL2 and reversed protein expression during the process of EMT. Importantly, by revealing the mechanism by which the CCL2-ERK1/2 pathway regulates epidermal cell migration during early wound formation under short-term hypoxic conditions, our findings help to elucidate the entire physiological process of wound healing and provide new promising targets for promoting wound repair in clinical practice.

## Methods

### Cell culture and hypoxia treatment

HaCaT cells were cultured in Dulbecco’s modified eagle medium (DMEM, HyClone, USA) supplemented with 10% antibiotics and 10% fetal bovine serum (FBS, OriCell, Uruguay). The cells were then incubated at 37°C under 5% CO_2_ and 95% humidity. Hypoxic conditions were created using a Forma Series II Water Jacket CO_2_ incubator (Thermo Scientific), in which the oxygen partial pressure was controlled at 1% and the temperature was 37°C.

### DNA microarray

HaCaT cells subjected to short-term hypoxia for different durations (0, 0.5, 1, 3 and 24 h) were collected. Total RNA was isolated, labeled and hybridized on the Clariom D platform following the manufacturer’s instructions (Affymetrix, Santa Clara, CA, USA). The original microarray data were deposited in the Gene Expression Omnibus database under access ID GSE132988. The research group previously conducted a detailed analysis of the data.

### Cell transfection

Plasmids and siRNAs were designed, synthesized and obtained from OLIGOBIO Corporation (Beijing, China) (Tables 2 and 3). HaCaT cells were added to 6-well plates (2 × 10^5^/well) and grown to 40–60% confluence. Then, Lipofectamine 2000 was used to transfect the cells with the appropriate shRNAs, and the cells were incubated for 8 h in serum-free medium. Afterward, the medium was exchanged for medium containing 10% FBS.

### Lentivirus production and infection

The establishment of lentiviral vectors harboring sh/OERNA-targeting CCL2 and the generation of HaCat cells stably expressing sh/OERNA were performed as previously described. The virus was generated with the pLVX-shRNA2-T2A-puro shRNA expression vector, the PCDH-CMV-MCS-EF1-copGFP-T2A-puro overexpression expression vector and the psPAX2 and pMD2 strains. The virus titers of sh-CCL2–1-plvx-shRNA2-mChrrry-T2A-Puro, sh-CCL2–2-plvx-shRNA2-mChrrry-T2A-Puro and sh-CCL2–3-plvx-shRNA2-mChrrry-T2A-Puro were 3.5 × 10^8^, 3.0 × 10^8^ and 4.0 × 10^8^ TU/ml, respectively. The virus titer of PCDH-CMV-CCL2-EF1-copGFP-T2A-puro was 2.5 × 10^8^ TU/ml (supplementary Figure S1, see online supplementary material). HaCaT cells were transfected with lentiviral particles harboring experimental or control vectors.

### Construction and validation of CCL2 interference and overexpression in HaCaT cells

Stable cell lines with CCL2 interference and overexpression were constructed with a lentiviral system. The shRNA coding sequences of the three targets were customized for CCL2 interference and the cDNA sequences were customized for CCL2 overexpression. The above sequences were transfected into 293 T-engineered cells via vector plasmids. Finally, the expression of CCL2 was verified at the transcriptional and protein levels. After the above steps, a stable cell line with CCL2 interference or overexpression was constructed and the resulting cell line was used for subsequent research.

### Wound scratch assay

HaCaT cells were inoculated in 6-well plates. The cells were then incubated with medium containing mitomycin C (10 μg/ml) for 2 h to reduce cell proliferation. After the cells reached confluence, a cell scratch was created with a 1000 μl plastic pipette tip, after which the cells were placed in the hypoxic chamber. Images of the scratch wounds were recorded at 0, 3, 6, 12 and 24 h using an inverted microscope (Olympus, Japan).

### Cell counting kit-8

HaCaT cells in the logarithmic growth phase were removed from the cell culture medium, washed with phosphate-buffered saline (PBS) three times, digested with prewarmed trypsin, centrifuged and extracted. The cell concentration was subsequently calculated with a cell counting plate. HaCaT cells were plated on a 96-well plate at 1x10^6^/ml and eight duplicate wells were established for each group. Afterward, 200 μl of cell suspension and 300 μl of PBS were added to each well, which prevented evaporation loss during subsequent experiments. An overnight incubation was performed to ensure adhesion. After the turbid supernatant was discarded, the cells were washed three times with 300 μl of PBS and then cultured in a cell incubator with 1% O_2_ for 0, 3, 6, 12 and 24 h. After hypoxic incubation, 10 μl of cell counting kit-8 (CCK-8) reagent was added to each well. After continuing to incubate for 1.5 h, the absorbance at 450 nm was determined with a microplate reader.

### Establishing and recording an acute full-thickness cutaneous wound rat model

With the permission of the Animal Welfare and Ethics Committee of the Army Medical University, male Sprague–Dawley rats weighing 200 ± 20 g were purchased from Beijing Vital River Laboratory Animal Technology Co., Ltd, and kept with a normal circadian rhythm following the 3R principle and free access to sufficient food and water. The rats were conventionally raised for 1 week in the Experimental Animal Center of Xin Qiao Hospital under specific pathogen-free conditions before the experiment. After the rats were anesthetized by intraperitoneal injection of pentobarbital sodium (32.5 mg/kg), a full-thickness cutaneous wound 18 mm in diameter was painlessly created on the back of each rat. Subsequently, the rats were divided into an FR1802024 group and a control group based on the application of an ERK inhibitor (FR180204; Beyotime, Shanghai). FR180204 was injected into the wounds of the FR1802024 group, and a digital camera was used to record the wound area at the same focal distance on days 0, 3, 6, 9, 12 and 15. On days 3, 6 and 15, tissue was carefully collected from the wound area. Part of the tissue was fixed in 4% paraformaldehyde fixation solution, embedded in paraffin and crosscut into 4 μm thick slices for further staining, and the other part was frozen in liquid nitrogen.

Wound healing rates were calculated on the basis of the following formula: wound healing rate (%) = (*S*_0_ − *S*t)/*S*_0_ × 100%, where *S*_0_ indicates the original wound area and *S*t indicates the wound area on a certain day.

### Hematoxylin and eosin and Masson staining

At 3, 6 and 15 days after the establishment of the wound model, the same number of mice in each group were euthanized after anesthesia. The wound tissue was fixed with paraformaldehyde, embedded in paraffin and then sliced into 4 μm sections. All slices were dewaxed and rehydrated before staining. After dewaxing and rehydration, slices from days 3, 6 and 15 were stained with hematoxylin and eosin (HE) for histological analysis. Masson staining was also performed with a Masson’s trichrome staining kit (Solarbio, China) according to the manufacturer’s instructions to observe collagen deposition. These slices were observed and photographed at 40x and 200x magnification (BX63, Olympus, Japan).

### Immunohistochemistry

The paraffin-embedded tissue slices were immunohistochemically stained for Ki67. After dewaxing with xylene and washing in PBS for 10 min, the slices were immersed in 0.01 mmol citrate buffer (pH = 6) at 95°C for 15 min to retrieve antigens. Afterward, the slices were incubated with 3% hydrogen peroxide for 15 min and then blocked with goat serum at room temperature for 15 min. Anti-Ki67 antibody (1 : 100, 34 330, CST) was added, and the sections were incubated at 4°C overnight. After that, the slices were incubated with a goat anti-rabbit secondary antibody and then stained with a diaminobenzidine kit (PV9000, ZSGB-BIO, Beijing, China). Finally, the slices were observed and images were recorded by microscopy (BX63, Olympus, Japan) at 200× magnification.

### Enzyme-linked immunosorbent assay

HaCaT cells were routinely cultured. After hypoxic treatment, the cell supernatant was collected at 0, 3, 6, 12 and 24 h. Whole blood was drawn from the rats after 3, 6 and 15 days and centrifuged at 3000 rpm for 15 min, then the supernatant was collected. The expression levels of tumor necrosis factor (TNF)-α and CCL2 in serum and HaCaT cells were detected according to the instructions of the enzyme-linked immunosorbent assay (ELISA) kits.

### WB analysis

HaCaT cells were seeded into 6-well plates and subjected to different treatments for 1 day. Then, the samples were washed using ice-cold PBS, and lysis solution was added to disintegrate the proteins and protease inhibitor cocktail (Selleck, Shanghai). Skin tissue (30 mg) was minced and homogenized. Then, the samples were centrifuged at 4°C and 13,000 rpm for 30 min. The protein concentration was determined by using a Bicinchoninic Acid Assay kit (BIOGROUND, Chongqing). Protein (50 μg) was separated via 10% sodium dodecyl sulfate (SDS)–polyacrylamide gel electrophoresis and transferred to polyvinylidene difluoride membranes (Millipore, Bedford, MA, USA). After the membranes were blocked for 2 h using 5% nonfat milk powder (Beyotime, Shanghai), they were incubated with primary antibody (dissolved in Tris Buffered Saline with Tween 20) overnight at 4°C and then incubated with secondary antibody for 1.5 h at room temperature. The immunosignals were identified using an enhanced chemiluminescence detection system (BIOGROUND, Chongqing). β-Actin and GAPDH, which served as loading controls, were identified and visualized. Specific information on the providers of the primary and secondary antibodies is as follows: N-cadherin rabbit pAb (22018–1-AP, 1 : 300, ProteinTech), E-cadherin rabbit pAb (20874–1-AP, 1 : 500, ProteinTech), Snail rabbit mAb (13099–1-AP, 1 : 500, ProteinTech), anti-TWIST antibody (bs-2441R, 1:300, Bioss), anti-Slug antibody (ab27568, 1:500, Abcom), phospho-p44/42 MAPK (Erk1/2) (Thr202/Tyr204) (D13.14.4E) XP® rabbit mAb (4370, 1 : 1000, CST), phospho-MEK1/2(Ser217/221) (41G9) rabbit mAb (9154, 1 : 500, CST), WAVE-2 (D2C8) XP® rabbit mAb (3659, 1 : 1000, CST), phospho-STAT3 antibody (AF3293, 1:500, Affinity), GAPDH (D16H11) XP® rabbit mAb (5174, 1 : 1000, CST), β-actin rabbit mAb (13E5) (4970S, 1:1000, CST), and anti-rabbit IgG, HRP-linked antibody (7074P2, 1 : 1000, CST).

### Statistical analysis

All the data used in this study were independent and repeated at least three times. One-way or two-way Analysis of Variance (ANOVA) with repeated measures followed by a Bonferroni *post hoc* test with *p*-value correction was used for data analysis. *P* values < 0.05 were considered to indicate statistical significance. All the data are presented as the means ± SDs and were analyzed using Prism 8 (GraphPad Software, La Jolla, CA, USA).

## Results

### Early short-term hypoxia increases CCL2 expression at the transcriptome level in HaCaT cells

**Figure 1:**
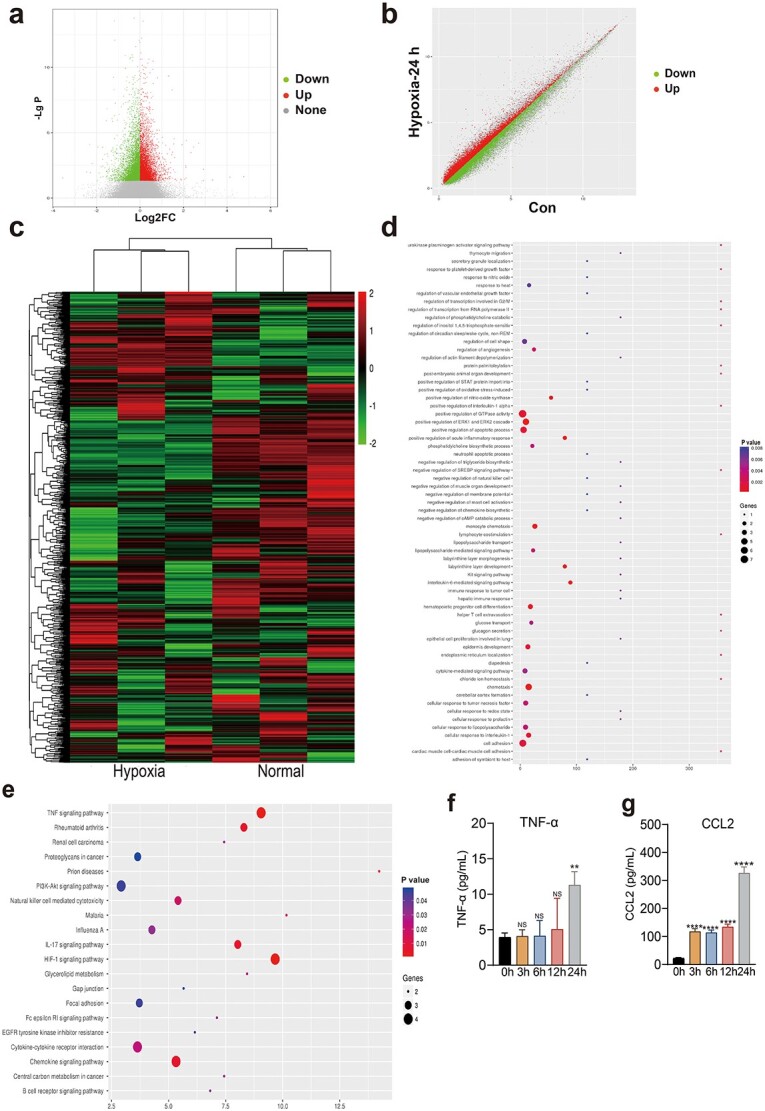
Genetic changes in HaCaT cells after 24 h of hypoxia. (**a**) Distribution of up- and downregulated genes after 24 h of hypoxia in the volcano plot. (**b**) Distribution of up- and downregulated genes after 24 h of hypoxia. (**c**) Differential expression of representative hypoxic and normoxic genes. (**d**) GO functional enrichment analysis of genes after 24 h of hypoxia. (**e**) Transcriptome expression after 24 h of hypoxia. (**f**) TNF-ɑ expression after 0, 3, 6, 12, and 24 h of hypoxia, as determined via ELISA. (**g**) CCL2 expression after 0, 3, 6, 12, and 24 h of hypoxia, as determined via ELISA. The data are presented as the mean ± standard deviation. ^*^^*^p < 0.001, ^*^^*^^*^^*^p < 0.0001; NS, no significant difference between groups. *GO* Gene Ontology, *TNF-α* tumor necrosis factor alpha

Short-term hypoxia can promote cell migration, which has been confirmed in relevant studies [36]. To further study the mechanism of hypoxia-induced HaCaT cell migration, we used a gene chip system to explore the effect of different hypoxia treatment durations on the expression of the HaCaT cell transcriptome. The volcanic map results demonstrated a significant difference in gene expression between the hypoxic group and the control group (Figure 1a). The scatter diagram and heatmap revealed that each differential gene exhibited good clustering (Figure 1b, c). Through GO enrichment analysis of the expression of cell migration- and adhesion-related genes, CCL2 was found to increase most significantly after 24 h of hypoxia treatment (Figure 1d, Table 1). In addition, KEGG pathway enrichment analysis suggested that the TNF-α signaling pathway plays an extremely crucial role in hypoxia regulation, which was also investigated (Figure 1e). ELISA revealed that CCL2 and TNF-α levels increased most significantly after 24 h of hypoxia (Figure 1f, g).

**Table 1:** GO enrichment analysis at 24 h of hypoxia compared with 0 h

**GO name**	**Gene symbol**	***P*** ** value**
Chemotaxis	FER	1.97425E-05
Chemotaxis	MAP2K1	1.97425E-05
Chemotaxis	CCL2	1.97425E-05
Chemotaxis	CCL20	1.97425E-05
Chemotaxis	PLAUR	1.97425E-05
Positive regulation of ERK1 and ERK2 cascade	IL6	0.000125718
Positive regulation of ERK1 and ERK2 cascade	MAP2K1	0.000125718
Positive regulation of ERK1 and ERK2 cascade	CCL20	0.000125718
Positive regulation of ERK1 and ERK2 cascade	CCL2	0.000125718
Positive regulation of ERK1 and ERK2 cascade	FBXW7	0.000125718
Monocyte chemotaxis	IL6	0.000206271
Monocyte chemotaxis	CCL2	0.000206271
Monocyte chemotaxis	CCL20	0.000206271
Positive regulation of nitric-oxide synthase biosynthetic process	CCL2	0.000590841
Positive regulation of nitric-oxide synthase biosynthetic process	CCL20	0.000590841
Cellular response to interleukin-1	CCL20	0.001042226
Cellular response to interleukin-1	CCL2	0.001042226
Cellular response to interleukin-1	IL6	0.001042226

*GO* Gene Ontology, *ERK* extracellular signal-regulated kinase, *IL* interleukin, *MAPK* mitogen-activated protein kinase

### Early short-term hypoxia promotes the migration and proliferation of HaCaT cells

**Figure 2:**
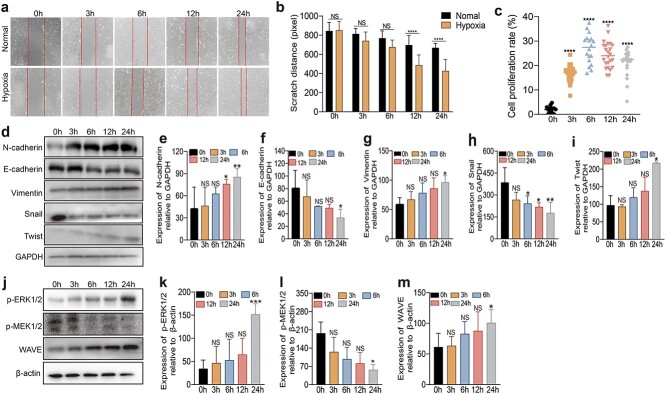
HaCaT cell migration and proliferation under hypoxia at different time points. (**a**) Cell scratch assays of the hypoxic and normoxic groups at 0, 3, 6, 12, and 24 h. (**b**) The width of the scratches was measured by ImageJ. (**c**) The proliferation of HaCaT cells at 0, 3, 6, 12, and 24 h was assessed by a cell counting kit-8 assay. (**d–i**) Expression of migration-related proteins. N-cadherin, E-cadherin, vimentin, Snail and Twist were detected via WB at 0, 3, 6, 12, and 24 h under hypoxia. (**j–m**) Expression of the ERK1/2 pathway-related proteins p-ERK1/2, p-MEK1/2 and WAVE at 0, 3, 6, 12, and 24 h determined by WB under hypoxia. The data are presented as the mean ± standard deviation. ^*^p < 0.05, ^*^^*^p < 0.01, ^*^^*^^*^p < 0.001, ^*^^*^^*^^*^p < 0.0001; NS, no significant difference between groups. *ERK* extracellular signal-regulated kinase, *WB* western blot

To verify the effect of hypoxia on HaCaT cell migration, we tested HaCaT cell migration under different hypoxic conditions (0, 3, 6, 12 and 24 h) through cell scratch assays, where the results indicated that, under short-term hypoxia (within 24 h), cell migration ability increased with time (Figure 2a, b). The proliferation ability of HaCaT cells under different hypoxic conditions (0, 3, 6, 12 and 24 h) was observed through CCK-8 experiments. The results suggested that, compared with that in the 0 h group, the proliferation ability was enhanced by hypoxia, within which the 6 h group exhibited the most remarkable increase in proliferation ability. The 24 and 3 h groups were relatively similar (Figure 2c).

Next, we focused on verifying whether cell migration under hypoxic conditions was related to the ERK1/2 pathway and mediated by regulating the EMT process. WB results demonstrated that, compared to those in the 0 h group, the expression of N-cadherin, vimentin and Twist, which are typical features of mesenchymal cells, was significantly greater, while the expression of E-cadherin and Snail suggested the loss of typical characteristics of epithelial cells, which was reduced in a time-dependent manner (Figure 2d–i). Moreover, through WB, the expression of p-ERK1/2, p-MEK1/2, and WAVE was also detected in HaCaT cells cultured under the same conditions. Compared with those in the 0 h group, the expression of p-ERK and WAVE was significantly greater, while the expression of p-MEK was reduced in a time-dependent manner, which was consistent with previous results, confirming the correlation between cell migration and the ERK1/2 pathway (Figure 2j–m) [37, 38].

### Construction and validation of CCL2-overexpressing and scrambled cells

**Figure 3:**
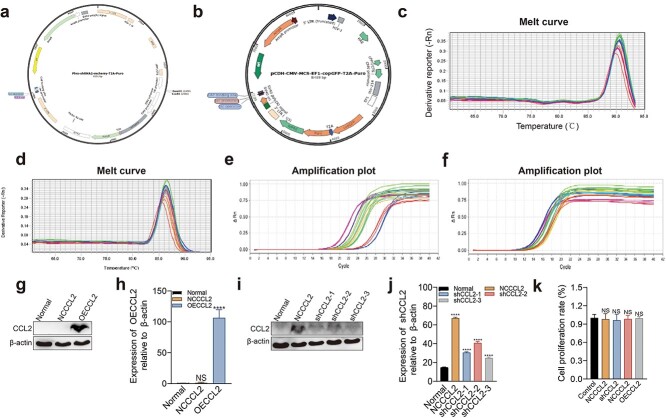
Construction and validation of CCL2-overexpressing and scrambled shRNA-treated cells. (**a**) Interference with the engineered plasmid mapping profiles. (**b**) Atlas of the overexpressed engineered plasmid. (**c**) Actin melting curve. (**d**) CCL2 melting curve. (**e**) Actin amplification curve. (**f**) CCL2 amplification curve. (**g**, **h**) Expression of the CCL2 protein in the overexpression groups. (**i**, **j**) Expression of the CCL2 protein in the interference group. (**k**) Cell proliferation in the interference and overexpression groups. The data are presented as the mean ± standard deviation. ^*^^*^^*^p < 0.001, ^*^^*^^*^^*^p < 0.0001; NS, no significant difference between groups

Stable transgenic cell lines with CCL2 interference or overexpression were constructed through a lentiviral system (Figure 3a–f). The shRNA coding sequences of the targets were customized for CCL2 interference (Table 2) and the cDNA sequences were customized for CCL2 overexpression (Table 3). The above sequences were individually transfected into 293 T engineered cells via a vector plasmid. Then, the expression of CCL2 was verified at the protein level. The results showed that the interference group and overexpression (OE) CCL2 group had the greatest effect on the protein level, (Figure 3g–j). CCK-8 assays showed that there was no significant effect on cell proliferation after treatment (Figure 3k).

**Table 2:** Coding sequence of CCL2 interfering RNA

**ID**	**5′**	**Stem**	**Loop**	**Stem**	**3′**
sh-CCL2 -1-F’	gatccg	TCATAGCAGCCACCTTCATTC	ctcgag	GAATGAAGGTGGCTGCTATGA	TTTTTT
sh-CCL2 -1-R’	aattAAAAAA	TCATAGCAGCCACCTTCATTC	ctcgag	GAATGAAGGTGGCTGCTATGA	cg
sh-CCL2 -2-F’	gatcc	GCTCGCGAGCTATAGAAGAAT	ctcgag	ATTCTTCTATAGCTCGCGAGC	TTTTTT
sh-CCL2 -2-R’	aattAAAAAA	GCTCGCGAGCTATAGAAGAAT	ctcgag	ATTCTTCTATAGCTCGCGAGC	g
sh-CCL2 -3-F’	gatccg	CCCAGTCACCTGCTGTTATAA	ctcgag	TTATAACAGCAGGTGACTGGG	TTTTTT
sh-CCL2 -3-R’	aattAAAAAA	CCCAGTCACCTGCTGTTATAA	ctcgag	TTATAACAGCAGGTGACTGGG	c

**Table 3:** CCL2 overexpression cDNA coding sequence

**ID**	**Sequence**
CCL2	gaattcgccaccATGAAAGTCTCTGCCGCCCTTCTGTGCCTGCTGCTCATAGCAGCCACCTTCATTCCCCAAGGGCTCGCTCAGCCAGATGCAATCAATGCCCCAGTCACCTGCTGTTATAACTTCACCAATAGGAAGATCTCAGTGCAGAGGCTCGCGAGCTATAGAAGAATCACCAGCAGCAAGTGTCCCAAAGAAGCTGTGATCTTCAAGACCATTGTGGCCAAGGAGATCTGTGCTGACCCCAAGCAGAAGTGGGTTCAGGATTCCATGGACCACCTGGACAAGCAAACCCAAACTCCGAAGACTTGAggatcc

### Early hypoxia promotes cell migration through the upregulation of CCL2

**Figure 4:**
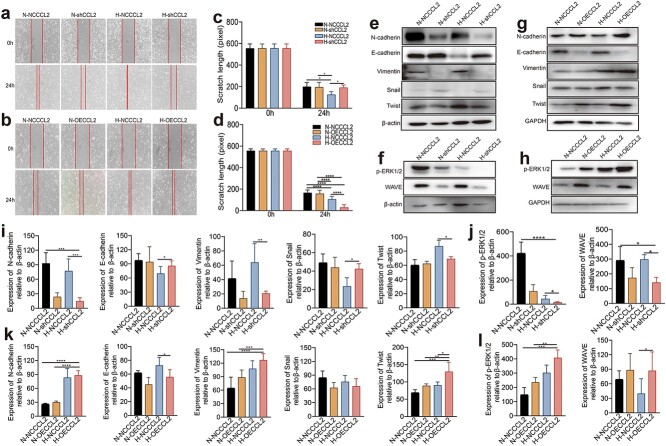
HaCaT cell migration after interference and overexpression of CCL2. (**a**, **b**) Cell migration of groups with disrupted and overexpressed CCL2 after 24 h of hypoxia. (**c, d**) The width of the scratch was measured by ImageJ software. (**e**) The expression of the migration-related proteins N-cadherin, E-cadherin, Vimentin, Snail, and Twist determined by WB after interference with CCL2. (**f**) Expression of the ERK1/2 pathway-related proteins p-ERK1/2 and WAVE determined by WB after interference with CCL2. (**g**) The expression of the migration-related proteins N-cadherin, E-cadherin, Vimentin, Snail, and Twist determined by WB in cells overexpressing CCL2. (**h**) The expression of the ERK pathway-related proteins p-ERK1/2 and WAVE determined by WB in cells overexpressing CCL2. (**i, j, k, l**) Protein expression was measured by ImageJ software. The data are presented as the mean ± standard deviation. ^*^p < 0.05, ^*^^*^p < 0.01, ^*^^*^^*^p < 0.001, ^*^^*^^*^^*^p < 0.0001

A cell scratch test was used to explore the effect of interference or overexpression of CCL2 on the migration ability of HaCaT cells after 24 h of hypoxia. The results in Figure 4 demonstrate that there was no significant difference between the control group (NCCCL2, infected with negative lentivirus) and the silenced group (shCCL2) under normoxia, while shCCL2 significantly impaired migration under hypoxia, which suggests that early hypoxia promoted cell migration through upregulating CCL2. The EMT process was also confirmed by WB, which revealed a significant decrease in the expression of mesenchymal cell-specific proteins, including N-cadherin, vimentin and Twist, while the expression of epithelial cell-specific proteins, such as E-cadherin and Snail, increased. Silencing CCL2 decreased the expression of ERK pathway-related proteins, such as p-ERK1/2 and WAVE.

The same experiments were then conducted to compare the control group and the OECCL2 group. Moreover, there was no significant difference between the control group and the OECCL2 group under normoxia, while OECCL2 markedly enhanced migration under hypoxia, which further confirmed that early hypoxia promoted cell migration through the upregulation of CCL2. Compared with the results of previous studies in which CCL2 was silenced, the WB results revealed the opposite trend in which the expression of N-cadherin, vimentin and Twist increased, while that of E-cadherin and Snail decreased. Overexpression of CCL2 led to an increase in p-ERK1/2 and WAVE.

### Early hypoxia promotes cell migration through the ERK1/2 pathway

**Figure 5:**
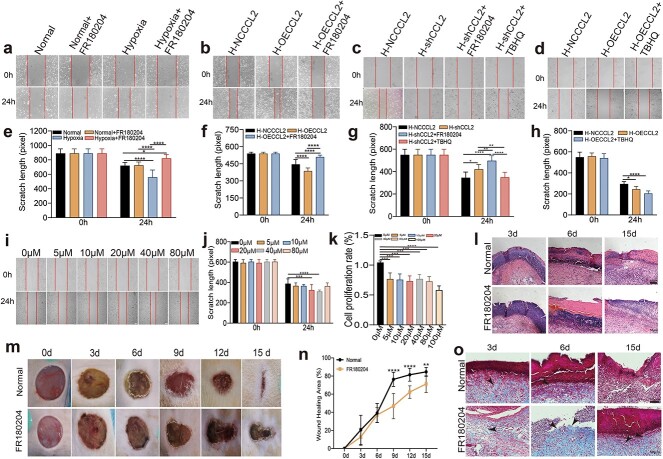
Early hypoxia promotes cell migration through the ERK1/2 pathway. (**a**) A cell scratch assay was used to detect the effect of FR180204 on migration under 24 h of hypoxia. (**b**) A scratch healing assay was used to evaluate the migration ability of OECCL2 cells after FR180204 treatment. (**c**) A scratch healing assay was used to evaluate the migration ability of shCCL2 cells after FR180204 and TBHQ treatment. (**d**) Cell scratch healing assay showing the effect of TBHQ treatment on the migration ability of OECCL2 cells. (**e–h**) The width of the scratch was measured by ImageJ software. (**i**) Migration capacity of HaCaT cells treated with different concentrations of TBHQ. (**j**) The width of the scratch was measured by ImageJ software. (**k**) The effect of TBHQ on the proliferation of HaCaT cells was determined by a cell counting kit-8. (**m**) Representative images of the healing process at 0, 3, 6, 9, 12 and 15 days. (**n**) The wound healing rate was measured at 0, 3, 6, 9, 12 and 15 days, and the error bars indicate the SDs; n = 6. (**l**, **o**) Representative plot of HE and Masson staining of trabecular tissue from rats in each group at 3, 9 and 15 days. The data are presented as the means ± standard deviations. ^*^p < 0.05, ^*^^*^p < 0.01, ^*^^*^^*^p < 0.001. Collagen deposition is indicated by black arrows

To further verify the regulatory effect of the ERK1/2 signaling pathway on the migration ability of HaCaT cells in a short-term hypoxic microenvironment, we used FR180204 (an ERK inhibitor) in subsequent cell scratch assays. According to the instructions, we selected 10 μM as the concentration of FR180204. As shown in Figure 5, the FR180204 group showed no significant difference from the control group under normoxia, while under hypoxia, the FR180204 group exhibited a decreased migration ability, which suggested that short-term hypoxia might promote cell migration through the ERK1/2 pathway.

Considering the previous experimental results that revealed that overexpression or silencing of CCL2 caused an increase or decrease in the expression of downstream ERK pathway-related proteins, we then focused on exploring the relationship between CCL2 and the ERK1/2 pathway. Next, we used FR180204 and an agonist (TBHQ) for further exploration. We screened the optimal concentration of TBHQ (40 μM) based on the scratch test and CCK-8 results. The final results demonstrated that FR180204 obviously impaired the migration of HaCaT cells overexpressing CCL2 under hypoxia. However, the application of TBHQ could to some extent offset the impact of CCL2 knockout on cell migration while enhancing the migration ability of HaCaT cells overexpressing CCL2 under hypoxic conditions. This evidence indicated that short-term hypoxia promoted wound healing by upregulating upstream CCL2 and subsequently upregulating the downstream ERK1/2 pathway.

### ERK inhibitor impairs wound healing in a full-thickness cutaneous wound rat model

To further investigate the role of the ERK1/2 pathway in wound healing, rats were first randomly divided into a control group and an FR180204 group. A full-thickness cutaneous wound 18 mm in diameter was then created on the shaved backs of the rats. FR180204 was injected into the wounds of the FR180204 group once every 3 days beginning at 0 days. Images of the wound area at 0, 3, 6, 9, 12 and 15 days are shown in Figure 5, where the FR180204 group exhibited a significantly slower wound healing rate than the control group.

HE staining was then used to explore the histopathological changes in the wounds. According to the HE sections, at 3, 6 and 15 days, the main inflammatory cells in the control group were neutrophils, while they were lymphocytes in the FR180204 group. Three days after the injury, new capillaries could be observed in the control group; however, in the FR180204 group, the opposite trend was observed. In both groups, the fibroblasts were irregularly arranged, skin appendages were not generated and there was still a large number of inflammatory cells. On day 6, there were markedly fewer inflammatory cells than before and more neatly arranged fibroblasts in the control group; however, there were still a few irregularly arranged fibroblasts, many inflammatory cells and newly emerged capillaries in the FR180204 group. On day 15, in the control group, new capillaries were more neatly arranged, inflammatory cells were significantly reduced and skin appendages such as sebaceous glands were generated. However, there were still many inflammatory cells, disordered fibroblasts and discontinuous epidermis. The Masson results demonstrated decreased collagen deposition in the FR180204 group and increased and dense collagen fibers in the control group.

**Figure 6:**
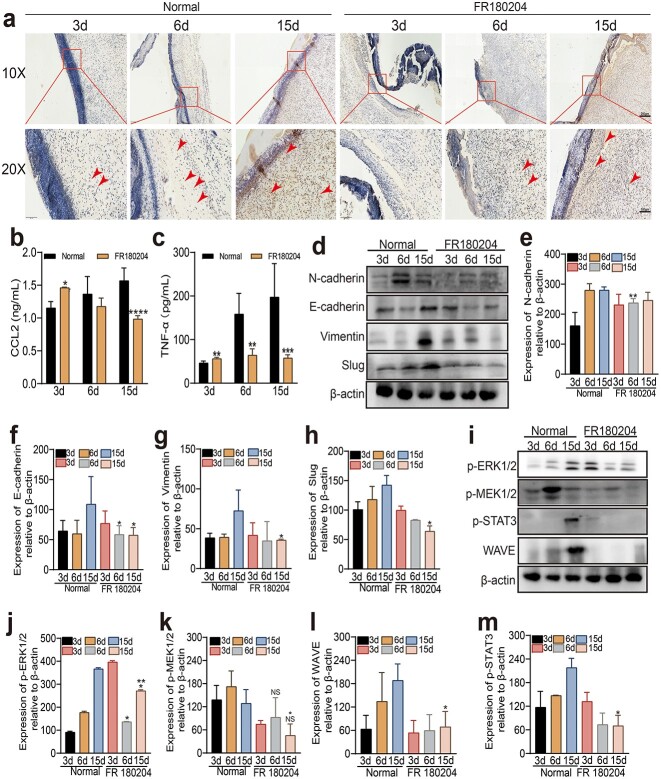
FR180204 inhibited wound healing. (**a**) Representative plot of immunohistochemical staining of Ki-67 in trauma tissue at 3, 9 and 15 days. (**b**, **c**) CCL2 and TNF-α expression in the serum of rats aged 3, 9 and 15 days determined by ELISA analysis. (**d–h**) The expression of the migration-related proteins N-cadherin, E-cadherin, Vimentin and Slug in the wound tissues was determined by WB at 3, 9 and 15 days. (**i–m**) The expression of p-STAT3 and the ERK pathway-related proteins p-ERK1/2, p-MEK1/2, and WAVE in the wound tissue was determined using WB at 3, 9 and 15 days. The data are presented as the means±standard deviations. ^*^p < 0.05, ^*^^*^p< 0.01, ^***^p < 0.001, ^****^p < 0.0001; NS, no sigsnificant difference between groups. The arrow indicates Ki-67^+^ cells. *TNF-α* tumor necrosis factor alpha, *ERK* extracellular signal-regulated kinase

Immunohistochemical staining was used to detect Ki-67-positive (Ki-67^+^) cells in the wound tissue of the FR180204 group. As shown in Figure 6a, there were fewer Ki-67^+^ cells in the FR180204 group at 3 and 6 days, and the number of Ki-67^+^ cells in the control group continuously decreased over time. Interestingly, the number of Ki-67+ cells increased, which suggested that inhibition of the ERK1/2 pathway delays the proliferation phase of wound healing. ELISA was used to detect the expression of CCL2 and TNF-α in wound tissue, which demonstrated that, compared with that in the control group, the expression of CCL2 and TNF-α increased after 3 days, while after 6 and 15 days, it was reduced (Figure 6b, c). These findings suggested that CCL2 responded to the short-term hypoxic environment on the wound surface; however, with the continuous application of inhibitors in the later stage, its expression gradually decreased.

The expression of EMT-related proteins in the tissue was also detected by WB. E-Cadherin and Slug expression decreased after 15 days, while after 3 and 6 days, there was no significant difference, indicating the transdifferentiation of epithelial cells into mesenchymal cells. N-Cadherin and vimentin were found to decrease on days 6 and 15, respectively, indicating a decrease in the acquisition of cell migration ability through EMT (Figure 6d–h). Downstream proteins related to the ERK1/2 pathway were also detected through WB and were significantly suppressed in the later stage of wound healing (Figure 6i–m).

## Discussion

As the first line of defense for the human body against external harmful factors, the skin is also highly susceptible to damage. The primary goal of current clinical treatment is to promote wound closure, in which the migration of epidermal cells from the edge of the wound to the center is a speed-limiting step. Oxygen has been confirmed to be associated with cell proliferation and differentiation [39]. Many studies have focused on providing oxygen to accelerate wound healing by promoting cell proliferation and migration and angiogenesis [40, 41]. Nevertheless, what has always been ignored is that, in the early stage of wound formation, the microenvironment around the wound becomes hypoxic due to the impaired vasculature. Then, the hypoxic status of the wound is aggravated by the recruitment of inflammatory cells with high oxygen consumption, and the early hypoxic microenvironment switches the repair program [40,42–44].

Other researchers agree that hypoxia might be a strong stimulus to certain types of cells or during certain physiological processes. For instance, chondrogenesis of stem cells was enhanced under hypoxia [45–50]. There have also been intensive investigations on diverse cancers, where the MAPK/ERK signaling system participates in the proliferation, migration, metastasis and resistance of cancer cells to chemotherapy, which can be triggered by hypoxia and reactive oxygen species-mediated injury and signaling. [51–53]. Rapid tumor cell growth increases oxygen consumption and leads to different degrees of pathological hypoxia and consequent activation of hypoxia-inducible factors [54, 55]. In the hypoxic state, the overexpression of hypoxia-inducible factors and activation of downstream signaling pathways (including CD47, CXCR4 and MAPK/ERK) can mediate immune escape in many ways, such as through the release of many immunosuppressive growth factors and cytokines [56, 57], upregulation of the negative immune checkpoint V-set immunoregulatory receptor in colon cancer [58], and induction of programmed cell death 1/programmed cell death 1 ligand 1 for T-cell inhibition [59, 60] and CD47 for macrophage suppression [61].

However, studies on the exact mechanism through which hypoxia promotes cell migration during wound healing are still rare. Our previous work with a gene chip system revealed significantly increased CCL2 expression after 24 h of hypoxia treatment, and further GO and KEGG pathway analyses revealed that CCL2 was enriched in the ERK1/2 pathway. The CCL2 and ERK1/2 pathways have been proven to be tightly associated with cell migration, but the relationships between CCL2 and the ERK1/2 pathway and wound healing have yet to be explored. Therefore, in this study, we aimed to explore the hidden mechanism by which the CCL2-ERK1/2 pathway regulates epidermal cell migration and how epidermal cells acquire migratory ability during early wound formation under short-term hypoxic conditions.

CCK-8 and cell scratch assays were performed on cell groups subjected to different treatments to verify the effect of hypoxia on migration and proliferation, where the increased migration and proliferation rates indicated that under early short-term hypoxia, the proliferation and migration ability of HaCaT cells increased over time. The WB results revealed upregulated N-cadherin, vimentin and Twist, which indicates the acquisition of migratory ability, and downregulated proteins, including E-cadherin and Snail, which indicates the loss of epithelial features. These findings all confirmed the role of EMT. The expression of downstream proteins related to the ERK pathway, such as p-ERK1/2, p-MEK1/2 and WAVE, was also found to increase. Then, we induced the overexpression and interference of CCL2 in HaCaT cells via lentiviral transduction under different conditions. Cell scratch test results suggested that neither overexpressing nor silencing CCL2 affected migration under normoxia, while under hypoxia, overexpressing CCL2 enhanced migration, while silencing CCL2 impaired cell migration. Combined with the WB results, these findings revealed a positive correlation between CCL2 expression and cell migration through EMT; specifically, hypoxia-mediated cell migration in the early stage occurred through EMT through the regulation of CCL2 expression.

The ERK inhibitor FR180204 was subsequently utilized to explore the regulatory effect of the ERK1/2 signaling pathway on cell migration ability. The results showed that FR180204 markedly impaired the migration of HaCaT cells under short-term hypoxic conditions but not under normoxic conditions, which suggested that ERK1/2 is involved in the cascade through which short-term hypoxia regulates cell migration. We further verified the promotional effect of the CCL2-ERK1/2 pathway on cell migration under short-term hypoxia through further cell scratch tests with FR180204 on the transfected cell line.

In addition, we constructed an acute full-thickness cutaneous wound rat model and applied an ERK inhibitor to the wound. Wound healing was significantly delayed in the FR180204 group. HE staining of the FR180204 group showed increased inflammatory cell infiltration and disorderly arrangement of fibroblasts. Masson staining revealed increased collagen deposition and a more neat and dense arrangement. WB results demonstrated decreased N-cadherin and Slug expression, which indicated impaired cell migration ability in the FR180204 group. Immunohistochemical staining revealed significantly fewer Ki-67^+^ cells in the FR180204 group at 3 and 6 days but more Ki-67^+^ cells, suggesting that inhibition of the ERK1/2 pathway delays the proliferation phase. Interestingly, CCL2 and TNF-α levels were found to increase on day 3 in the FR180204 group but decrease on days 6 and 15, which was consistent with previous speculation that in the early stage of wound formation, hypoxia serves as a strong stimulus to trigger wound healing; therefore, CCL2 and TNF-α were upregulated to recruit inflammatory cells, fibroblasts, etc., and under continuous suppression of FR180204, the CCL2-ERK1/2 pathway was downregulated.

## Conclusions

Our work explored the precise role of the CCL2-ERK1/2 pathway and EMT in wound healing under early short-term hypoxia. The CCL2-ERK1/2 pathway responds to hypoxia in early wounds and turns on the switch for cell recruitment, during which epidermal cells acquire migratory ability through EMT and promote wound closure. These results will deepen our understanding of the regulation of cellular pathways in the early microenvironment of wounds, fill in the gap in knowledge and reveal new potential targets for clinical practice.

## Abbreviations

CCK-8: Cell counting kit-8; ERK1/2: Extracellular signal-regulated kinase 1/2; ELISA: Enzyme-linked immunosorbent assay; EMT: Epithelial–mesenchymal transition ; MAPK: Mitogen-activated protein kinase; PBS: phosphate-buffered saline; TFF1: Trefoil factor family 1; WB: Western blot.

## Supplementary Material

Supplemental_Figure_tkae017

## Data Availability

All data and related analyses are included in this published article. All other data is available from the corresponding author upon request.
