# Supplementary figures and images for: Early short-term hypoxia promotes epidermal cell migration by activating the CCL2-ERK1/2 pathway and epithelial–mesenchymal transition during wound healing
Source: Burns Trauma. 2024 Jun 17;12:tkae017. doi: 10.1093/burnst/tkae017 (PMC11182653; doi:10.1093/burnst/tkae017)

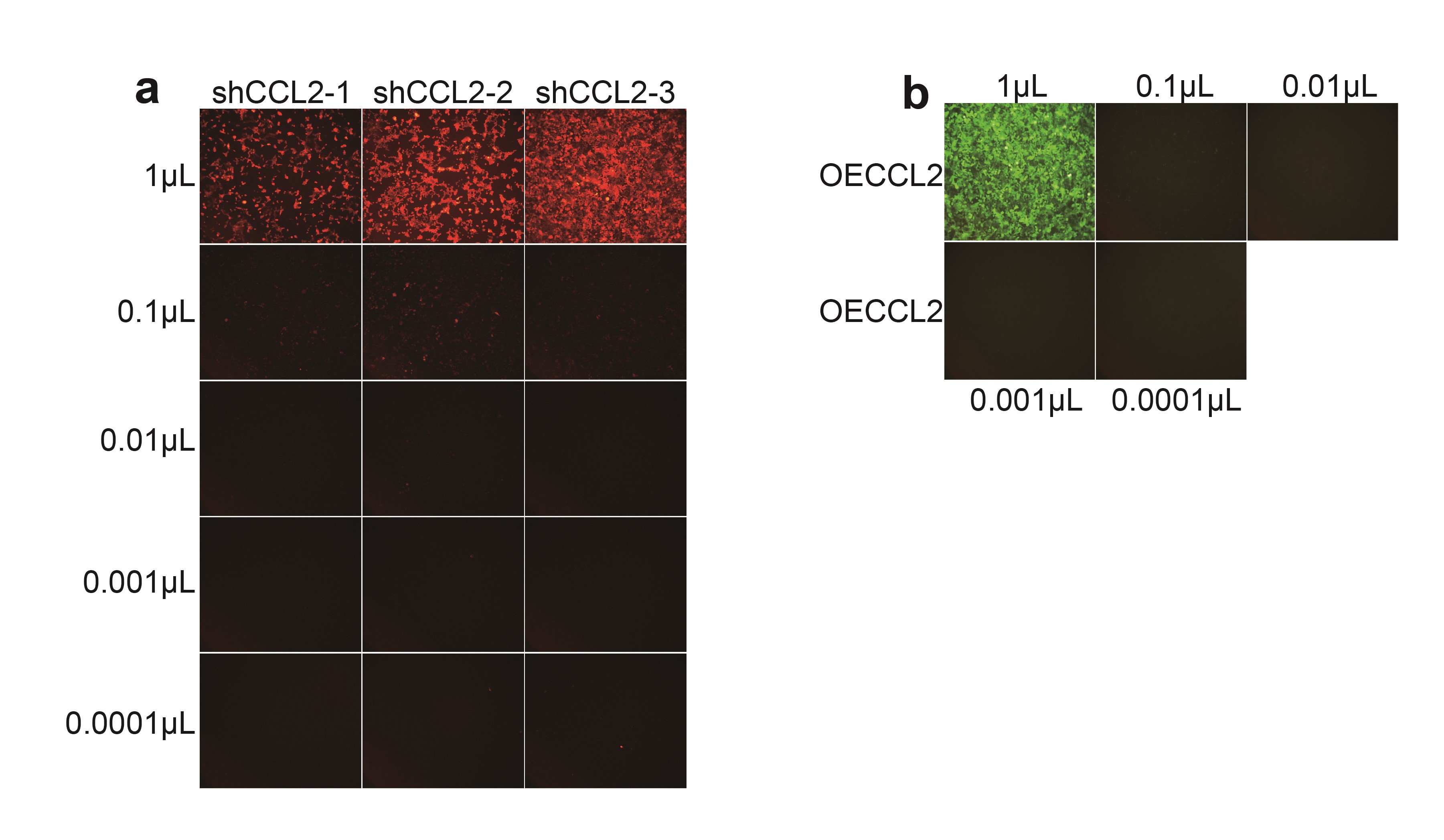

Supplement: Supplemental_Figure_tkae017 [file supplemental_figure_tkae017.jpeg]
